# Disentangling source of moisture driving glacier dynamics and identification of 8.2 ka event: evidence from pore water isotopes, Western Himalaya

**DOI:** 10.1038/s41598-020-71686-4

**Published:** 2020-09-18

**Authors:** Om Kumar, A. L. Ramanathan, Jostein Bakke, B. S. Kotlia, J. P. Shrivastava

**Affiliations:** 1 School of Environmental Sciences, Jawaharlal Nehru University (JNU), New Delhi, 110067 India; 2 Department of Geology, University of Delhi, Delhi, 110007 India; 3 Department of Earth Science and Bjerknes Centre for Climate Research, University of Bergen, Allègaten 41, 5007 Bergen, Norway; 4 Centre of Advanced Study in Geology, Kumaun University, Nainital, 263002 India

**Keywords:** Climate sciences, Environmental sciences

## Abstract

Two atmospheric circulation patterns, the Indian Summer Monsoon (ISM) and mid-latitude Westerlies control precipitation and thus glacier variability in the Himalaya. However, the role of the ISM and westerlies in controlling climate and thus past glacier variability in the Himalaya is poorly understood because of the paucity of the ice core records. In this article, we present a new Holocene paleorecord disentangling the presence of the ISM and mid-latitude westerlies and their effect on glacier fluctuations during the Holocene. Our new record is based on high-resolution multi-proxy analyses (δ^18^Oporewater, deuterium-excess, grain size analysis, permeability, and environmental magnetism) of lake sediments retrieved from Chandratal Lake, Western Himalaya. Our study provides new evidence that improves the current understanding of the forcing factor behind glacier advances and retreat in the Western Himalaya and identifies the 8.2 ka cold event using the aforementioned proxies. The results indicate that the ISM dominated precipitation ~ 21% of the time, whereas the mid-latitude westerlies dominated precipitation ~ 79% of the time during the last 11 ka cal BP. This is the first study that portrays the moisture sources by using the above proxies from the Himalayan region as an alternative of ice core records.

## Introduction

**Figure 1:**
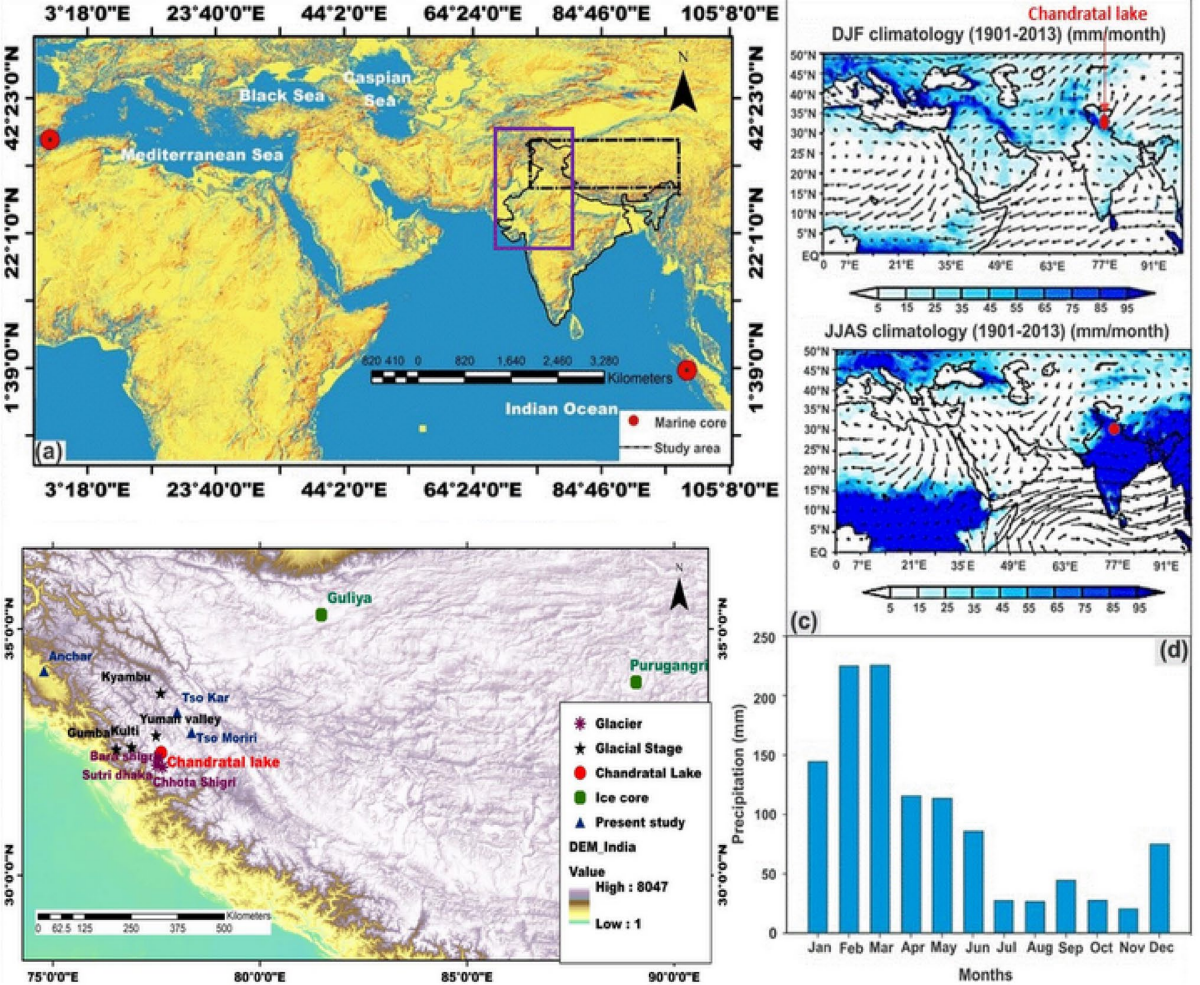
(**a**, **b**) Maps showing locations of study areas and earlier published sites of paleoclimatic study; (**c**) spatial distribution of precipitation during winter (DJF) and summer (JJAS) over the study regions (red dot indicates the location of Chandratal). Color shading shows JJAS and DJF monthly rainfall climatology(mm/month) during 1901–2013 (wind and precipitation data obtained from the National Center for Environmental Prediction/National Center for Atmospheric Research (NCEP/NCAR) and Global Precipitation Climatology Centre (GPCC) respectively; (**d**) monthly precipitation data from nearest Chhota Shigri glacier weather statio^57^ (note the stronger influence of Westerlies precipitation). Purple circle show indicates the Indus settlement and Block rectangle shows the location of archives including glacier valley. Map composed using Esri ArcGIS 10.4.

The Ice cores from Greenland and Tibet have been used to reconstruct past monsoon strength^1,2^ and various proxies have been applied to identify the past climatic events^3,4^. Because of the Westerlies and ISM circulation patterns, the climate of the Indian Himalaya is affected both by the Mediterranean and ISM as a source of precipitation (Fig. 1) and has a decisive influence on the socio-economic set-up of the region^5–9^. Existing glacier reconstructions from Himalaya are based on cosmogenic radionuclide dating (CRN) and optically stimulated luminescence (OSL) dating from different valleys, indicating that the Western Himalaya has experienced several glaciation stages in the past, e.g., the Chandra glaciation, Batal glaciation, Kulti glaciations and Sonapani glaciation in the past^10–12^. Although, adequate glacier chronologies have refined a number of glacial events over the last few years from different valleys of the Western Himalaya (i.e., Yunam valley, Miyar basin, and Karzok valley^13–15^), however, driver of the source of precipitation contributing to the mountain glacier advances is not well understood and remains a topic of debate^10–12^. Moreover, the scarcity of ice core records from the Indian Himalaya makes it difficult to understand the role of precipitation and temperature in the Quaternary glaciations. Since the d-excess values reflect westerly precipitation during winters in the Western Himalaya^16^, reconstruction of moisture sources during the Holocene from sediment records provides a new avenue for understanding past climate and glacier variability in the Himalaya. Reconstruction of dynamics of moisture changes during the Holocene is of great importance for societal development (e.g., Indus civilization) and future climate changes. Several studies suggest an intensification of Westerlies and reduced ISM leading to the deurbanization of Harappan civilization^5–9^. Hence, it is felt that the high-resolution multi-proxy records from parts of the Indus valley civilization areas appear vital to understand the role of ISM and mid-latitude Westerlies together by archeologists and paleoclimatologists.

Considering this, we have studied sediments from Chandratal lake (32°29′43″ N 77°36′48″ E, 4,300 m), formed during the Batal glacial stage^17^, and situated at the junction of mid-latitude Westerlies and ISM circulation (Fig. 1).

We also attempted to answer the following questions; (1) is the ice mass stored in the glaciers in the Western Himalaya formed by precipitation transported by mid-latitude Westerlies or the ISM and, (2) can pore water stable isotope records be trusted as a proxy to disentangle different Holocene climatic phases, including the 8.2 ka BP event?

**Figure 2:**
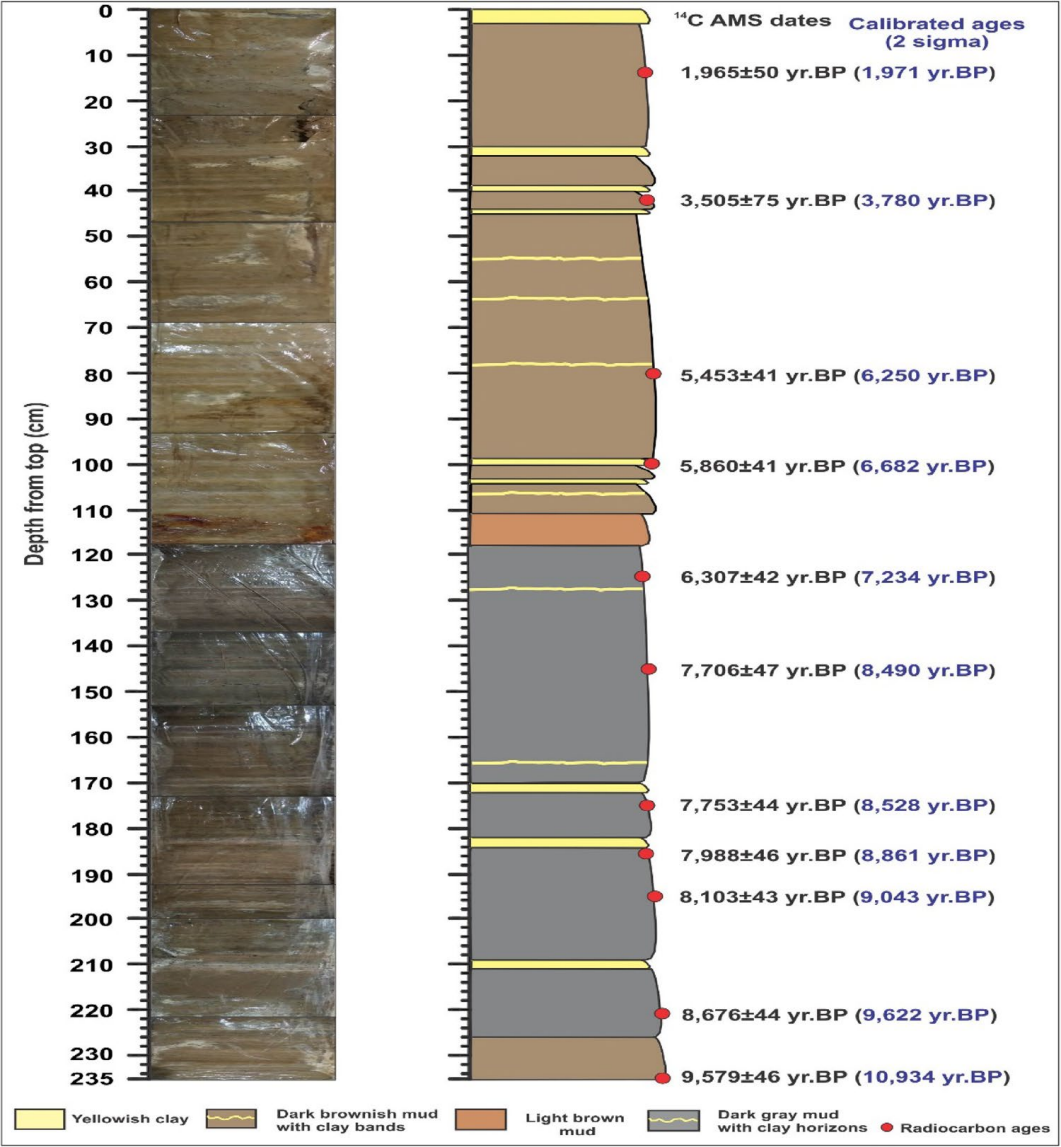
Photographic image and litholog of the Chandratal core with AMS radiocarbon dates and calibrated ages.

Under the project *Water-related effects of changes in glacier mass balance and river runoff in Western Himalaya, India: past, present and future* (GLACINDIA) (India–Norway) in 2015, we retrieved 27 sediment cores from different parts of the Chandratal using gravity corer (maximum 2 m) and piston corer (maximum 6 m) and selected one of the cores (235 cm long) to analyze using multi-proxy approach including porewater isotopes, mineral magnetism, grain size, and permeability, etc. The lake core (Fig. 2) consists mainly of brownish to blackish mud with cm-scale clay horizons. The sediments are micro-laminated without any sand lenses or bands and are generally homogenous in nature (Fig. 2). We extracted porewater samples at every 5 cm interval and analyzed for triple water isotopes by cavity ringdown spectroscopy (CRDS) using the Piccarotriple water isotope analyzer (L2140-i). Since the lake sediments contain a high percentage of impermeable clay which can retain different aspects of water isotope signatures to establish a strong basis for paleoclimatic reconstruction of the palaeo-monsoons in the Western Himalaya as the porewater isotopes (δD and δ^18^O) from lake Agassiz have already been successfully used to infer Late Pleistocene climatic oscillations^18^. The permeability of the sediments was calculated using the equation as permeability (k) = 760*d^2^e^(−1.31σi)^, where k is permeability, d is the mean grain size (mm) and σi is standard deviation in phi (ϕ).The increased fine grain size dust consists of low permeability and high porosity.The results reflect low-permeability in sediments, reflecting the potential to preserve past climatic signal.

The lake porewater line δD porewater is calculated as 4.66*1δ^18^O − 25.996 (R^2^ = 0.82, n = 49) and lake surface water line δD as 2.53*1δ^18^O − 57.48 (R^2^ = 0.51, n = 23) (Supplementary Fig. 1). The slope of pore water is 4.66 with an intercept as − 25.99. The Local meteoric water line (LMWL) generated from Sutri Dhaka glacier (snow, discharge and rainfall) is computed as δD = 7.51*δ^18^O + 8.88 (R^2^ = 0.9797, n = 88) (Fig. S1). The d-excess values of > 17‰ from Sutri Dhaka glacier in the nearby area, reveal the sources of moisture from the Mediterranean region^16^.The deuterium excess (d-excess) is a climatic independent indicator to track the source of moisture and is defined as d (‰) ≡ δD − 8.δ^18^O^19^ and controlled by wind speed, relative humidity and temperature from the moisture-generating reservoir^20,21^. The d-excess is used to delineate the source of precipitation and its low values during the ISM reflect moisture transport from the Indian Ocean^16,22^ whereas high values show moisture from the Westerly precipitation^16,23^.Therefore, we have used the same d-excess value to trace the origin of moisture source either from the ISM or the Westerlies. Located at the junction of the Westerlies and ISM, the Chandratal receives sediment through catchment erosion, weathering, and windblown dust. The back trajectories and d-excess sources provide evidence whether the moisture originates from the Mediterranean region and picking the dust particles and redistribute it to the Western Himalaya during winters (Fig. S3). Besides isotopes proxy, we have used grain size and magnetic measurements to understand the precipitation intensity and climatic processes in the surrounding catchments. For further understanding of the records, we have applied wavelet analysis to decompose the time series. The wavelet analysis has been used to identify solar cyclicity and applied to various archives^24^.

## Results

**Figure 3:**
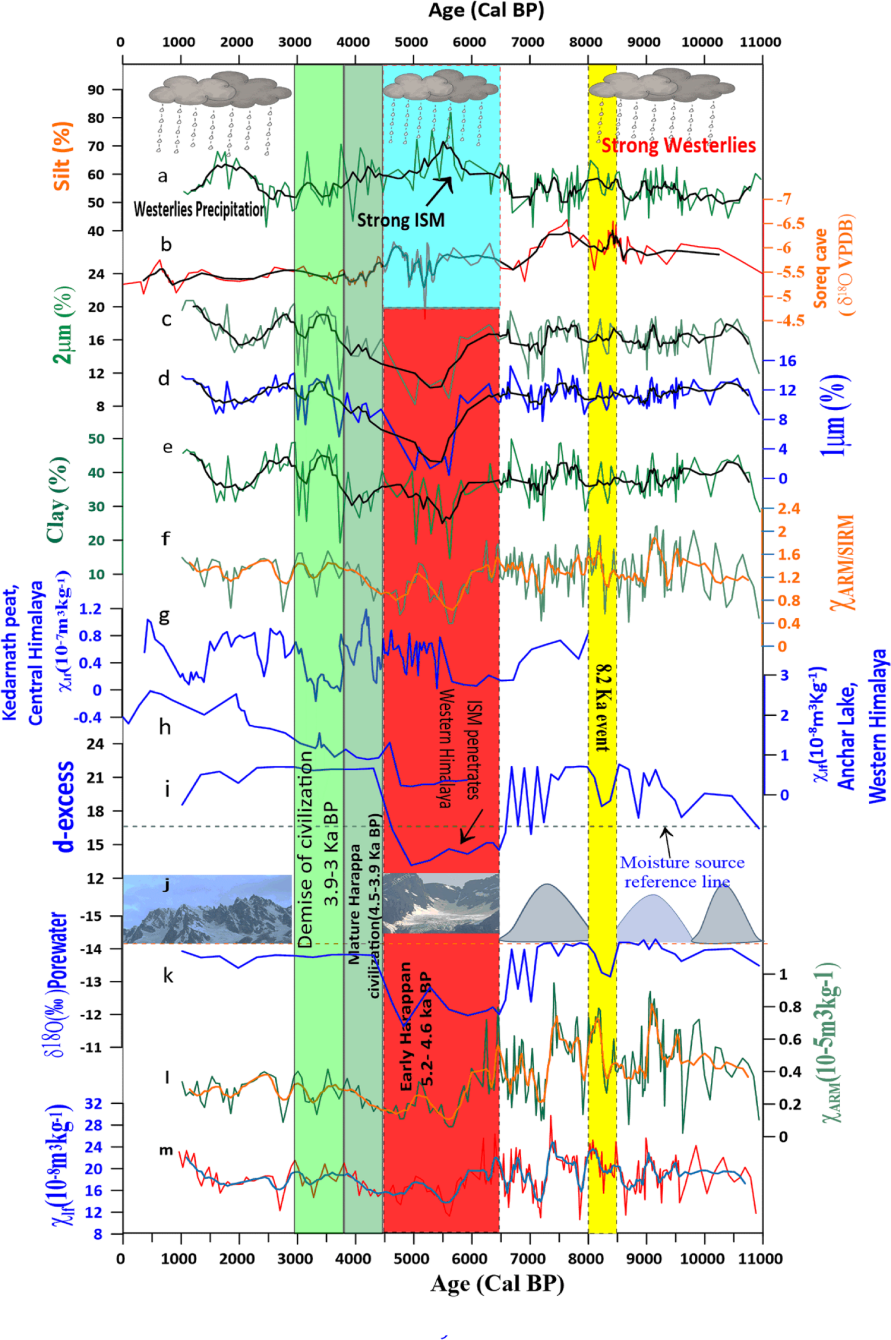
Comparison of Chandratal lake sediment results with other paleoclimate records from ISM and Westerlies dominated regions. (**a**) Percentage of silt; (**b**) westerlies representing δ^18^O (‰) values from Soreq cave^53^; (**c**) percentage of dust particle size (2 μm); (**d**) percentage of dust particle size (1 μm); (**e**) percentage of clay, (**f**) χ ARM/SIRM; (**g**) magnetic susceptibility records from Kedarnath^43^; (**h**) χ_lf_ from Anchar lake^39^; (**i**) porewater d-excess parameter; (**j**) dashed line delineates sources of moisture (below 17 per mil reflects ISM) and Schematic diagram representing glacier fluctuations due to mid-latitude Westerlies and ISM precipitation; (**k**) porewater δ^18^O (‰) value from Chandratal lake (present study); (**l**) χ_ARM_ (10^–5^ m^3^ kg^−1^) and (**m**) magnetic susceptibility (χ_lf_ 10^–8^ m^3^ kg^−1^). Figure prepared using Grapher 13 (Golden Software LLC) (www.goldensoftware.com).

The results of the multi-proxy analyses of the Chandratal lake core are shown in Fig. 3. We generated the record covering the last 11,000 cal BP (hereafter ka BP) record of the ISM and mid-latitude Westerlies using δ^18^Oporewater stable isotopes from Channdratal lake sediments and identified presence of ISM and tracked the 8.2 ka BP global event. Our results also cover the time span of evolution and the fall of Harappan civilization. The lake sediments mainly consist of detrital and autogenic origin and the fine-grained dust (1 and 2 μm) may be considered to have carried by the Westerly winds^25^ (Fig. S3). Since the grain size is widely used to reconstruct the monsoon intensity, we have also used grain size (percentage of silt and fractions of clay) proxy for comparison with d-excess proxies to disentangles paleoclimatic conditions. Based on previous studies, meteorological data from Chhota Shigri glacier station, Back trajectories and d-excess value suggest the dominance of westerlies (Fig. 1; Supplementary Figs. 1, 2, 3). Therefore we assume fine-grain dusts are mainly transported by westerlies and this further confirmed by the intensification of Westerlies in the region (Fig. 1). The < 4 μm grain size indicates the Westerly wind circulation and shows a positive correlation with the Soreq cave δ^18^O record (Fig. 3). During these periods, with the dominance of Westerlies, the region experienced cold and dry air which is not favorable for the growth of vegetation as compared to the case in the Central Himalaya which is characterized by the intensified ISM during summer. Therefore, Our interpretation is that the dominance of ISM may be interpreted as leading to an increased supply of total organic carbon (TOC), which is anti-correlated with magnetic susceptibility (MS). Further, a high percentage of silt and total organic carbon combined with low values of MS and d-excess represents the presence of the ISM in the region. The porewater isotopic composition of the studied lake core is similar to the bottom water isotopic effect during the time of deposition^26^.The pore water extracted from clay-rich glacial till marine and lacustrine sediments have been used to understand paleoclimate and paleoenvironmental conditions^27–29^. Therefore, the high percentage of clay considered as the potential to preserve the isotopic signal during Holocene (Fig. 3). Thus, we compared our results with mid-latitude Westerlies, represented by the Soreq speleothem δ^18^O and other archives, dominated by mid-latitude-westerlies as well as the ISM (for details, see Fig. 3).

The Chandratal sediments are characterised by high variability in sand, silt and clay content.. The early Holocene (11.0–10.0 ka BP) sediments are represented by sand (7.5%), silt (52.7%), and clay (39.6%).The percentage of grain size particle is calculated as 1 μm (average = 12, Standard Deviation (SD = 1.4) and 2 μm (average = 16.3, SD = 2.13. The MS values range from 15.4 to 22.0 (average 18.3), χ_ARM_ ranges from 0.23 to 0.55 (mean values 0.39) and χ_ARM/SIRM_ varies from 0.67 to 1.79 with an average of 1.10 from 11 to 10 ka BP (see Fig. 3). However, at a depth between 175 and 160 cm (during 8.2 ka BP), the sand varies from 1.3 to 12.17% (average 5.1%), silt from 52.5 to 62% (mean 57.5%), and clay between 30.9 and 43% with an average of 37.4%. The average value of 2 μm particle is 16.6% (SD = 1.5) and a mean value of 1 μm is 11.2 (SD = 1.13).

From 9.6 to 7.36 cal BP, the d-excess values range from 17.4 to 22.4‰ with an SD of 1.56, and the δD values range from − 86.2 to − 92.8‰ with an average of − 91.2‰. The MS value varies from 10.96 to 23.83 with an average of 17.92, the χ_ARM_ ranges from 0.09 to 0.61 with an average of 0.36 and the χ_ARM/SIRM_ ranges from 0.42 to 2.09 with an average of 1.36 (SD = 0.35). The percentage of silt ranges from 41.59 to 64.87 with an average of 54.72 (SD = 5.12), that of clay varies from 28.63 to 48.73 with an average of 38.42 (SD = 4.15), that of a 1 μm particle ranges from 8.72 to 14.91 with an average of 11.54 (SD = 1.56) and that of a 2 μm particle varies from 11.86 to 18.82 with an average of 16.28 (SD = 4.15).

From 7.36 to 6.5 cal BP, the magnetic susceptibility values range from 10.96 to 23.83 with an average of 17.92, the χ_ARM_ value ranges from 0.09 to 0.61 with an average of 0.36 (SD = 0.13) and the χ_ARM/SIRM_ value ranges from 0.44 to 1.71 with an average of 1.30 (SD = 0.29). The percentage of silt varies from 5.12 to 64.87 with an average of 54.13, that of clay varies from 4.15 to 49.85 with an average of 37.86 (SD = 5.82) and those of 1 and 2 μm particles vary from 1.28 to 15.26 with an average of 11.38 (1.78) and from 1.56 to 19.49 with an average of 16.03 (SD = 2.35), respectively.

The mid-Holocene (6.5–4.5 ka BP), the sediments are dominated by sand (4.56%), silt (62.5%), and clay (32.9%) with average 2 μm values as 13.5% (SD = 3.5) and 1 μm as 6.9% (SD = 4.8). The d-excess values range from 13.1 to 16.3 (average 14.7), δ^18^O‰ values from − 11.6 to − 12.8‰ (average − 12.2‰) with δD values ranging from − 80 to − 89‰. During this period, the enriched values of δ^18^O and low d-excess (below 17‰) (see Fig. 3) suggest that the moisture was transported from a shorter distance (apparently Indian ocean) (Fig. 3i), whereas, more depleted values of δ^18^O during mid-Holocene reflect that the moisture was transported from far distance (i.e., Mediterranean region; Fig. 3i). From 6.5 to 4.5 ka BP, susceptibility values range from 11.27 to 26.39 (average 17.42), χ_ARM_ from 0.06 to 0.8 (average 0.28; SD = 0.17) and χ_ARM/SIRM_ from 0.40 to 1.84 (average 1.10). The silt varies from 50.09 to 81.88% with mean values of 62.28 (SD = 6.92) and clay ranges from 14.62 to 42.58 (average 33.05). The fraction of 1 and 2 μm particles vary from 0.42 to 12.86 average as 7.54 (SD = 4.22) and 8.22–17.82 with mean as 13.97 (SD = 3.12) respectively.

From 4.5 to 3.9 ka BP, the susceptibility values range from 14.9 to 19.5 with an average of 16.9 (SD = 1.75), χ_ARM_ from 0.16 to 0.31 with a mean of 0.22 (SD = 0.06) with χ_ARM/SIRM_ varying from 0.79 to 1.42 with an average of 1.12 (SD = 0.21). The silt fraction varies from 43.36 to 69.35% with a mean of 59.15 (SD = 7.11) and clay ranges from 26.6 to 40.5% with an average of 33.0 (SD = 9.08). A fraction of 1 μm is recorded as 7.61–10.2% with a mean of 9.1% (SD = 1.0) and of 2 μm is observed as 13.7–16.1% with average values of 14.8 (SD = 1.0).

From 3.9 and 3.0 ka BP,the susceptibility values range from 14.71 to 21.54 (average 18.6) , χ ARM from 0.14 to 0.41 having mean value as 0.28 (SD = 0.09) and χ_ARM/SIRM_ ranges from 0.86 to 1.66, averaging as 1.37 (SD = 0.22). The sand varies from 0.4 to 20.13% with average values as 20.1 (SD = 4.4%) and clay from 34.51 to 48.68%, averaging as 40.56 (SD = 4.56). The 1 μm fraction ranges from 5.7 to 14.3% with an average of 11.6% (SD = 2.0), whereas, 2 μm fraction lies between 10.03 and 20.41% with mean values as 17.2% (SD = 2.0).

From 3.0 ka BP to present, the susceptibility values are recorded from 12.3 to 23.3 with an SD of 3.22, χ_ARM_ ranges from 0.09 to 0.4 and χ_ARM/SIRM_ ranges between 0.66 and 1.54 (average 1.31; SD = 0.06). The silt ranges from 52.90 to 68.04% with average values 58.51% (SD = 5.57) and the clay ranges between 31.19% and 46.77% with an average value as 40.36% (SD = 6.04). Similarly, 1 μm and 2 μm values range from 8.79 to 13.86% with an average of 11.69 (SD = 1.90) and 14.49–20.75% with a mean of 18.42 (SD = 2.11) respectively.

## Discussion

The timings and duration of glacier fluctuations in Himalaya have been dated and reconstructed using cosmogenic radionuclides (CRN) and optically stimulated luminescence (OSL) dating techniques. We argue that depleted δ^18^O value and high d-excess values of δ^18^Oporewater during the Kulti glacier advance (11–10 ka)^11^, Gumba glacier advance (10–8 ka)^13^, Yunam valley glacier advance (7.9 ± 1–6.9 ± 0.9 ka)^14^ and Kyambu glacier advance in diversified valleys of Himalaya took place due to the increased precipitation, transported by the mid-latitude Westerlies. During these glacier events, the d-excess value is greater than 17, as demarcated by the blue dashed line (see Fig. 3), indicating that moisture comes from the Westerlies.

Between 11.0 and 10.0 ka BP, precipitation is slightly lower as shown by δ^18^Oporewater and low value of percent TOC in the Chandra peat^30^ and Tso Kar lake sediments^31^ due to cold dry air, transported by the Westerlies. This is followed by enhanced precipitation during Gumba glacier advance as is reflected by the more depleted value of δ^18^Oporewater and high values of χ_lf,_ Silt (%), χ_ARM_ and χ_ARM/SIRM_ and also high Ti content in the Tso Kar lake, reflecting high runoff input into the lake in the Western Himalaya^31^. Higher (> 17) d-excess values and decreased TOC percentage in the Chandra peat^30^ confirm the precipitation due to the Westerlies.

From 9.6 to 7.36 ka BP, the d-excess values range from 17.4 to 22.4‰ with SD of 1.56, and δD value ranges from − 86.2 to − 92.8‰ (average − 91.2‰). Following this there is a period, with slightly reduced precipitation between ~ 8.5 and 8.0 ka BP as recorded in the δ^18^Oporewater proxy. Between 8.5 and 8.3 ka BP decreased values of χ_lf,_ χ_ARM,_ and χ_ARM/SIRM_ are observed in our record. This suggests an anomaly during the 8.2 ka event which is also reflected in the porewater values (see Fig. 3).This anomaly is due to temperature changes and which lasted ~ 150 year are already discussed^1^ .Added by higher values of d-excess (> 17), it is suggested that Westerly precipitation were dominated in the region around 8.2 ka event. Based on d-excess proxy, we interpret that d-excess value above 17 per mil indicates moisture transported by mid-latitude westerlies and values below 17 per mil suggest air masses coming from Indian Ocean (see Fig. 3). From this point of view, more depleted value of δ^18^O during 8.2 Ka event may correspond to longer travel distance from Mediterranean air masses compared to the shorter distance from Indian Ocean where from ISM is transported. The isotope-based evidence reflects both the δ^18^O and d-excess have been punctuated during this event. Overall result suggests, higher d-excess values during the 8.2 Ka event might due to decreases in evaporation and shift in relative humidity at moisture source region. The mid-latitude Westerlies air moisture carrying dust particles can also be shown by the back trajectories HYSPLIT model (Supplementary Fig. 3). In the high altitude Himalaya, precipitation occurs in form of snow and starts nucleation around these dust particles with favorable growth environment (− 3 °C to – 30 °C)^38^ to develop various shapes of snow crystals, leading to deposition of aeolian dust particles in the lake.The permeability ranges from 0.001 to 0 01 with an average value of 0.005, reflects sediments is impermeable. Consequently, The high percentage of clay throughout the core shows that the porewater stable isotopes have the potential to precisely capture the past climatic events including the 8.2 ka event (Fig. 3).This porewater stable isotopes of clay-rich approach have been also applied by researcher to track the past climatic evenet^27–29^ Multiple archives and proxies have been used by various researchers to recognize 8.2 ka event, e.g., application of δ^18^O from the Greenland ice core and cave speleothems^32–34^ arboreal pollen in lake sediments^35^ and other multi-proxy records from Himalaya and northwestern India^5,36^.The air masses from Mediterranean, Caspian and Black seas travel a long-distance from the Mediterranean region, leading depleted δ^18^Oporewater and d-excess value above 17‰, during early Holocene, confirming the role of the Westerly precipitation in the Western Himalaya. This is further confirmed by .the presence of *Pinus sylvestris* pollen from Tso Kar lake in the nearby area also points to the strong influence of the Westerly winds in the region during 8.8–7.4 ka BP^37^. Therefore, we disagree with the suggestion^14^ that the early Holocene glacier advance was caused by the enhanced ISM.

Another significant event recorded in the Chandratal sediments, dated to 6.5–4.5 ka BP, is marked by a high percentage of silt, low values of χ_lf,_ χ_ARM_, and χ_ARM/SIRM_. This age bracket seems to indicate the presence of the ISM in the Western Himalaya. The δ^18^O porewater ranges from − 12.8 to − 11.63‰ with an average of − 12.2‰ (SD = 0.3), and d-excess varies from 13.2 to 16.2‰ with an average of 14.8‰. The isotopic δ^18^O porewater and d-excess reveal the moisture originating from the Indian Ocean brings warm and humid air which favors vegetation growth, leading to increased TOC concentration which showing anti-correlation with susceptibility values, as recorded in the Chandratal sediments. The d-excess values below 17‰ and enriched values of δ^18^O porewater may further strengthen the ISM contribution for glacier growth in the region, reflecting that the ISM had penetrated the Western Himalaya during 6.5–4.5 ka BP.The Westerlies based records from the region, e.g., high values of silt percentage and low values of MS from the Anchar lake sediments^39^ and other Westerlies based results, Trilokinath peat^40^. Puruogangri ice core^41^ are in agreement with our results. The Chandra peat records also show Holocene climate optima (6.7–5.7 ka BP), which covel with our results^42^.

The decrease in dust concentration particle size (1 and 2 μm) from 6.5 to 4.5 ka BP suggests that the atmospheric circulation changed from being westerlies dominated to the ISM (Fig. 3). This is further confirmed by the ISM dominated records, e.g., Kedarnath peat^43^ and Gujjar peat^44^, recording low magnetic susceptibility and high values of LOI. This intensified ISM coincides with Kedar glacial stage (~ 7 ka), Shivling glacial stage (~ 5 ka)^45^, Rajbank glacial advance (RBS 2) (6.1 ± 0.4 ka), Raj Bank stage (RBS 3) at 5.0 ± 0.5 ka^46^ and Tons valley in the Central Himalaya around ~ 5 ka^47^. These time windows correspond to the flourishing of early Harappan civilization in various parts of India including tributaries of Indus^6,8,48–50^. Based on δ^18^O, δD and d-excess supplemented with other proxy record^8^ researchers have observed high rainfall in the northwestern Rajsthan resulting in the growth of urban Harappan centre during its early civilization. Our results also suggest the intensified ISM may have provided a favorable climate to ecological changes and agriculture development. Our interpretation is that the aforementioned glacier advances in the central Himalaya are caused by ISM intensification.

During 4.5–3.9 ka BP, The average δ^18^O values − 13.7‰ and average d-excess value 21.3‰ together with enriched δ^18^O of Soreq stalagmite^53^ and Puruogangri ice core^41^, compared to the early Holocene reflect slightly decreased Westerly precipitation (Fig. 3). This was perhaps a transition phase when ISM circulation began swinging to the Westerlies as registered in the isotopic records and dust (Fig. 3). Based on the planktonic community structure^9^ have also inferred the presence of the winter Westerlies during 4.5–3.0 ka BP. a period belonging to mature Harappa civilization, the urban settlement density was increased in the Indus valley^48,50^. The archeological study suggests that the cold climatic condition favors the wheat and barley production, on which these populations depended^51,52^.

Between 3.9–3.0 ka BP, the δ^18^O value ranging from − 13.8 to − 13.7‰ with an average of − 13.8 and average d-excess value as 21.7(‰) reflect the Westerlies precipitation Based one isotopic evidence and highest dust activity (see Fig. 3) clearly indicates cold and dry climatic prevailed in the region. The records from Tso Moriri, Tso Kar^31^ Triloknath^40^ reflects a decreased in precipitation intensity in Westerlies and also evidence by other records^7,55,56^.This period corresponds to the fall of Harappa civilization and perhaps was responsible for deurbanization. Here, we interpret that during the time of the demise of Harappa civilization dust activity has increased northwestern India (Fig. 3).

After 3.0–1.0 ka BP, there is a slight increase in precipitation, as evidenced by the depleted values of δ^18^O (− 13.7‰).This high d-excess (21‰) indicates that the region experienced heavy precipitation dominated by the westerlies, and this also favors glacier advance Kyambu glacial stage (3.4–0.2 ka BP)^15^, Gomuche glacier stage (2.1 ± 0.4 ka BP)^14^, Stok valley glacier (~ 2.1 ± 0.9 ka BP)^14^ and Mentok Kangri valley glacier (1 ± 0.1 ka BP)^14^ in the Western Himalaya.

## Conclusion

We have presented a new Holocene record disentangling monsoon variability and the role of the ISM versus the mid-latitude westerlies on the basis of a multiproxy approach in which we compare our record with other paleoclimatic records from the region. Application of porewater isotope proxy, a unique method, has been used to quantify and disentangle the source of precipitation and its role in the glacier dynamics of the Himalaya and Indus valley civilization. Our findings suggest that the impact of Westerlies was stronger between ~ 11.0 and 6.5 ka BP and ~ 5.2–1.0 ka BP, signifying that this Westerly precipitation was the main driver of past glacier advance in the study area as well as in the Lahul Spiti valley including valley glaciers of Chandra basin (e.g., Bara Shigri glaciers), one of the biggest glaciers crafted as a result of the Westerlies.

However, around 6.0–4.5 ka BP, enriched δ^18^O and low d-excess values, supplemented by other paleoclimate records indicate ISM penetrated in the Western Himalaya, reflecting the ISM contribution to the valley glaciation in Central and Western Himalaya (Shivling and Kedarnath glacial stages) and providing favorable climate to the early Harappan population development. We also suggest that during this time, Chandra river discharge should have been increased and intensified, triggering landslide activity in the valley.

Our results also indicate that the porewater stable isotopes have the potential to capture past climatic events (e.g., 8.2 ka event) and d-excess can also be used as a new input proxy for climate model simulation in the Indian Himalaya. Our study may also attract various scientists, particularly Historians and Archaeologists to unravel the history as well as causes of the collapse of the Indus civilization in northwestern Himalaya. This is the first study that disentangles the moisture source (ISM or mid-latitude Westerlies) as an alternative to the ice core records from the Himalayan region.

## Methods

The meteorological data, obtained from Chhota Shigri glacier, the nearest weather station (see Fig. 1) show that about 75–80% precipitation in the study area is contributed by the Westerlies^56^. A total of 27 sediment cores were retrieved using piston corer under GLACINDIA project (2015)^57^ and a 235 cm long sediment core was selected for extracting porewater samples (at every 5 cm interval) using a syringe connected with 0.25 μm filter at glacier laboratory of Jawaharlal Nehru University (JNU), New Delhi. The samples were transferred to 2 ml vials and stored at 4 °C to avoid isotopic fractionation and each sample was injected five times to avoid memory effects. The triple water isotopes were analyzed by Piccaro cavity ringdown spectrometer (L2140-i) at JNU, New Delhi. The isotope values are reported as per mil (‰) versus corresponding standard as δ = (*R*_sample_/*R*_reference_ − 1) × 1,000 where R_sample_ is the ratio of ^18^O/^16^O or ^2^H/^1^H. The analytical precision for δ^18^O and δ^2^H was 0.09‰ and 0.45‰ respectively. All stable isotope compositions are reported using the standard delta notation (δ) and expressed in part per mil (‰) and the results were normalized with V-SMOW-SLAP scale by analyzing standards.

The analytical uncertainty of each sample (no replica) corresponds to the polled standard deviation (σ*p*) as;

$${S}_{pooled}= \sqrt{\frac{\left({n}_{1}-1\right){S}_{1}^{2} + \left({n}_{2}-1\right){S}_{2}^{2} +\cdots + ({n}_{k}-1){S}_{k}^{2}}{{n}_{1} + {n}_{2} +\cdots + {n}_{k} - k}}$$

where n is the number of replicas of each sample, S, the standard deviation of each group of the replica (4–5) and K is a total number of samples. The minimum reported uncertainty for δ^18^O (0.2‰), for δD (2‰)^21^ and some laboratories report lesser values (δ^18^O = 0.03, δ^2^H = 0.17)^58^.

We used air mass back trajectory for 144-h Hybrid single-particle Lagrangian Integrated Trajectory (HYSPLIT) model and required meteorological input was selected using archived data set from Air Resources laboratory^25^. The trajectory represents the air moisture sources and pathways to deliver dust to the study area (Fig. S3). The grain size data and magnetic susceptibility data were smoothened using Local Weighted Scatterplot Smoothing (LOWESS or LOESS) with bootstrap 95% confidence level (Fig. S2). LOWESS is a robust regression technique that permits a flexible curve by removing outliers and noise^59^. We also performed the wavelet analysis to get the spectral signature from the Chandratal lake sediment proxy archive (Fig. S4). The wavelet analysis (WA) method is suitable when because of non-stationary variability (i.e., discontinuities and frequency and magnitude changes)^60^.

The sediment core was sub-sampled at a 1 cm interval to obtain high-resolution grain-size data and sand, silt, and clay particle sizes (%) (n = 235) were determined using Laser Particle Size analyzer (Microtrac 3,500) at JNU, New Delhi. Eleven samples were analyzed for AMS ^14^C dating by using 500KV pelletron at Inter-University Accelerator Center (IUAC), New Delhi and Department of Geology, Radiocarbon laboratory, Solvegatan, LUND, Sweden (see Fig. 2). Before analysis, the samples (~ 1 g each) were treated using Acid Base Acid (ABA) protocol^61^. After removing carbonate, samples were neutralized by repeated washing with 18MΩMiliQ water and subsequently treated with 0.5 N HCl, and then with 1 N HCl, and finally the samples were freeze-dried overnight before graphitization^61^. The AMS ^14^C radiocarbon dates were calibrated using calib 7.1^62^.

## Supplementary information

Supplementary Information
