# Supplementary material for: Disentangling source of moisture driving glacier dynamics and identification of 8.2 ka event: evidence from pore water isotopes, Western Himalaya
Source: Sci Rep. 2020 Sep 18;10:15324. doi: 10.1038/s41598-020-71686-4 (PMC7501258; doi:10.1038/s41598-020-71686-4)
Supplement: Supplementary file 1 — Supplementary Information [file 41598_2020_71686_MOESM1_ESM.docx]

**Disentangling source of moisture driving glacier dynamics and identification of 8.2 ka event: Evidence from pore water isotopes, Western Himalaya**

**Om Kumar^1,2^*, AL. Ramanathan^1*^, Jostein Bakke^3^, BS.Kotlia^4*^, JP.Shrivastava^2^**

[alrjnu@gmail.com](mailto:alrjnu@gmail.com)*, [omkrs007@gmail.com](mailto:omkrs007@gmail.com), [bahadur.kotlia@gmail.com](mailto:bahadur.kotlia@gmail.com*)

^1^School of Environmental Sciences, Jawaharlal Nehru University, New Delhi-110067, India

^2^Department of Geology, University of Delhi-110007, India

^3^Department of Earth Science and Bjerknes Centre for Climate Research, University of Bergen, Allègaten 41, 5007, Bergen, Norway

^4^Centre of Advanced Study in Geology, Kumaun University, Nainital-263002, India

*Corresponding authors

Supplementary Fig. 1. Local meteoric water line based on lake water and glacier samples from Western Himalaya (different symbols are used for the glacier, pore water and surface water samples


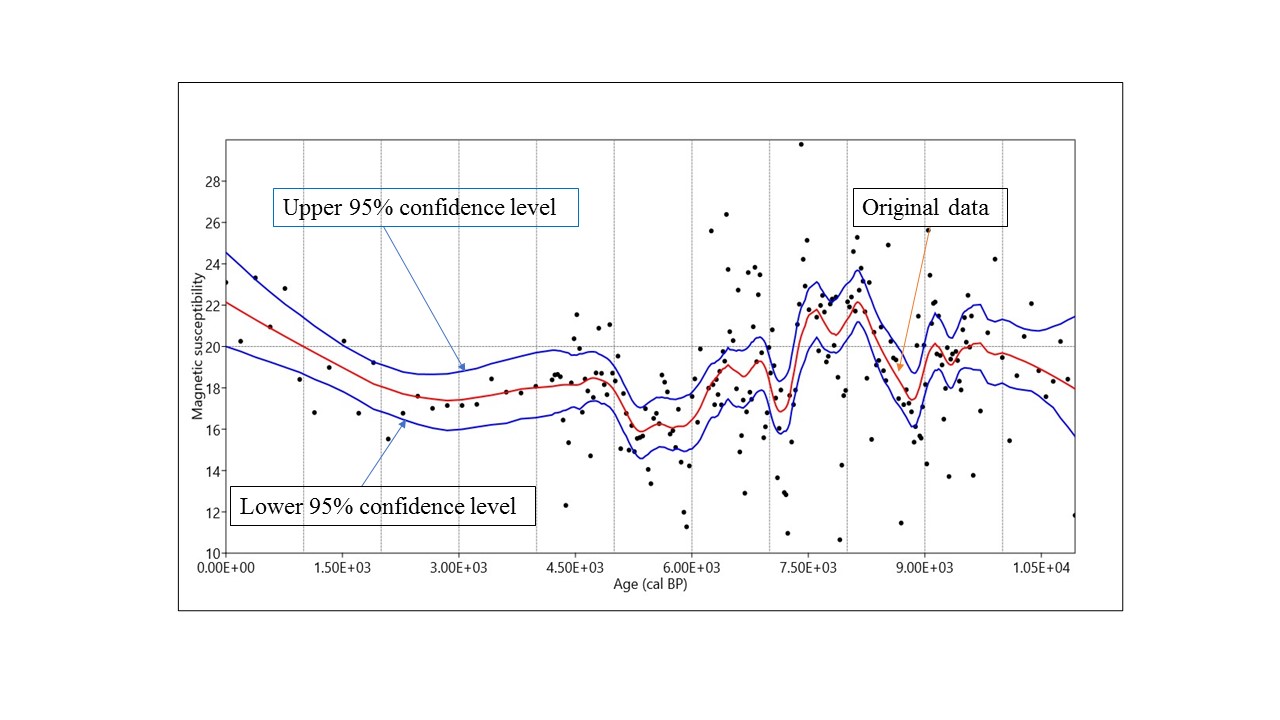


Supplementary Fig. 2a. Magnetic susceptibility data using LOWESS-smooth method with bootstrap 95% confidence level (upper and lower blue lines show 95% confidence level, and red line LOWESS smooth.


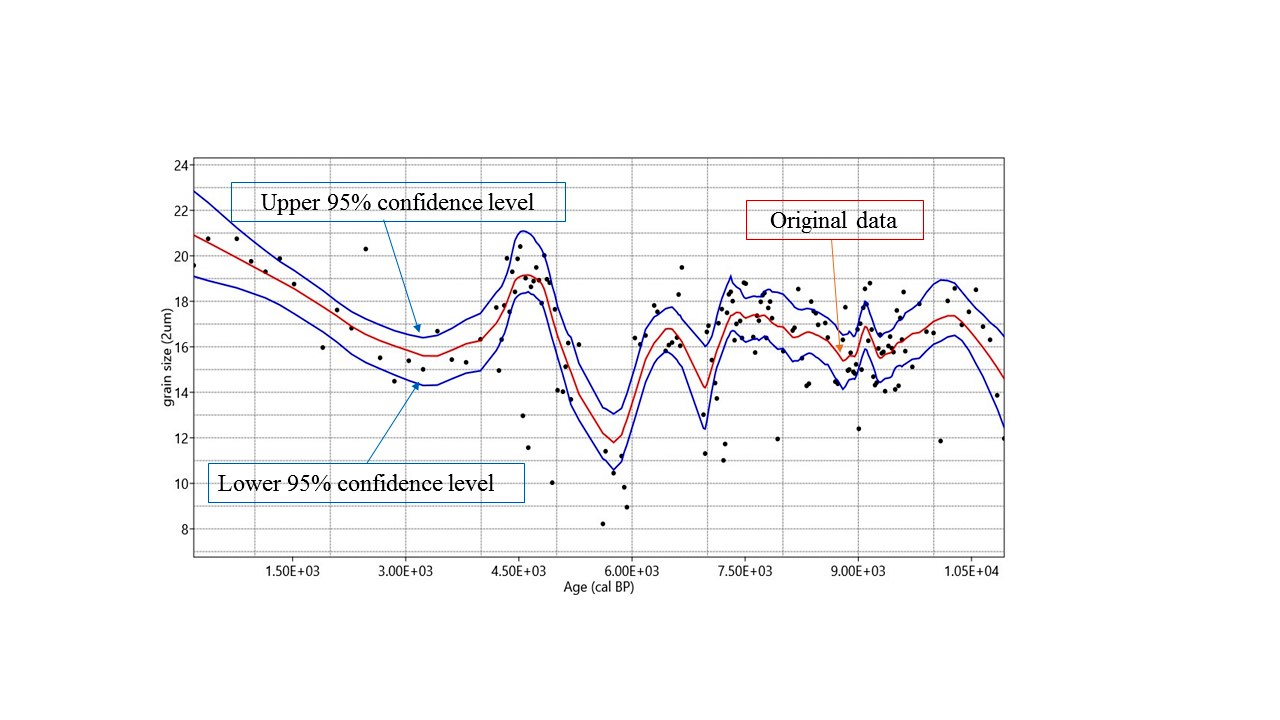


Supplementary Fig. 2b. Grain size (2µm) data using LOWESS-smooth method with bootstrap 95% confidence level (upper and lower blue lines at 95% confidence level and red line represents LOWESS smooth.


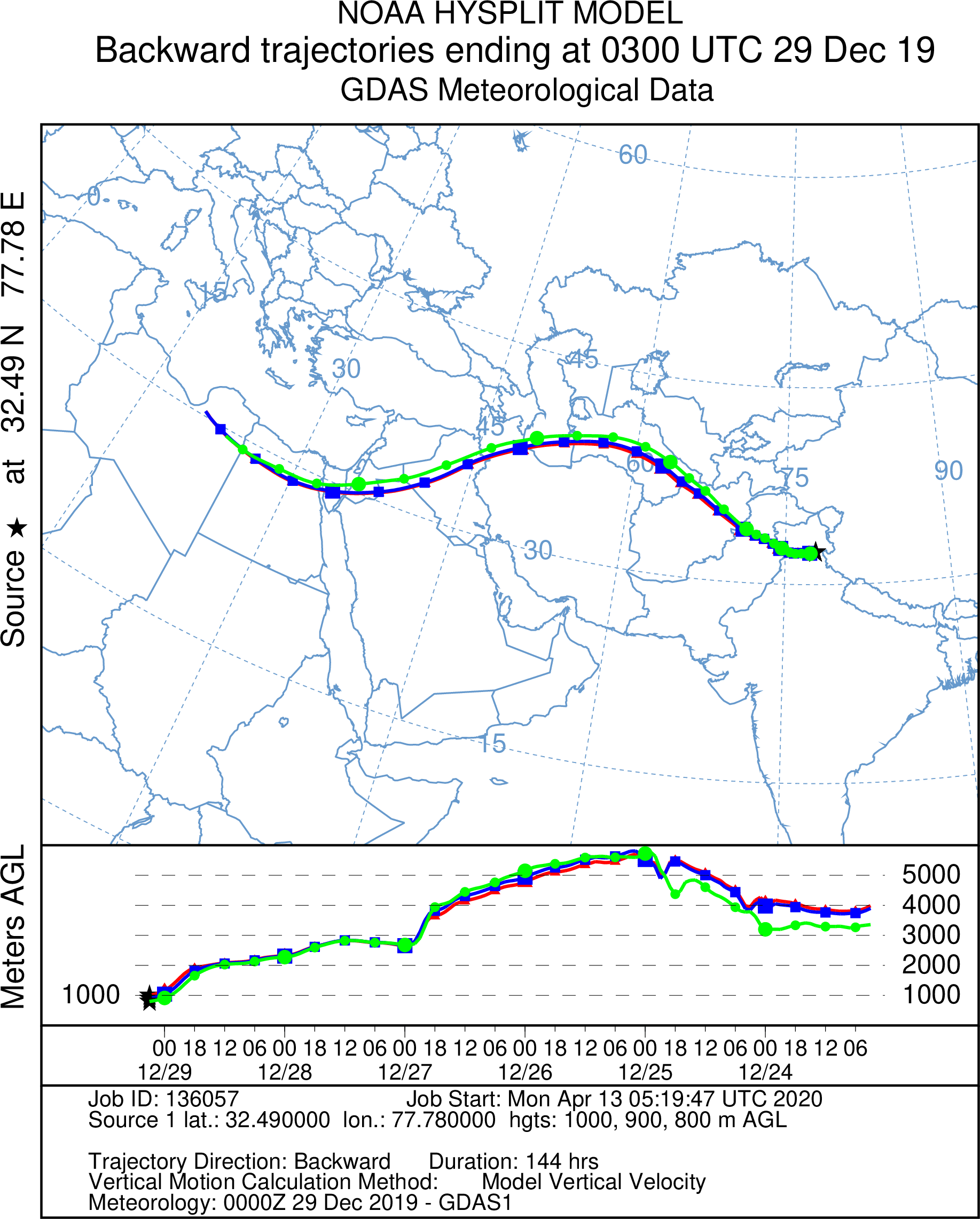


Supplementary Fig. 3. 144-hHYSPLIT back trajectories reflecting air moisture originating from Mediterranean sea and crossing over arid region and redistributing dust to the study area during winter season.The map is generated using HYSPLIT (HYbrid Single -Particle Lagragian Integrated Trajecory) Model Version 4.2.0 (<https://www.arl.noaa.gov/>).


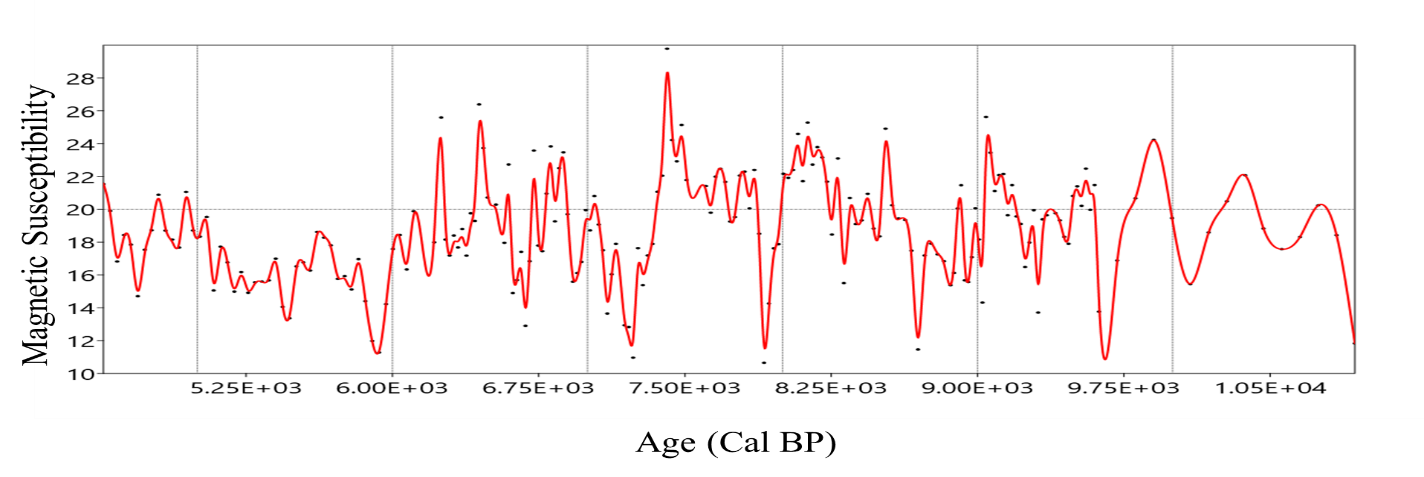

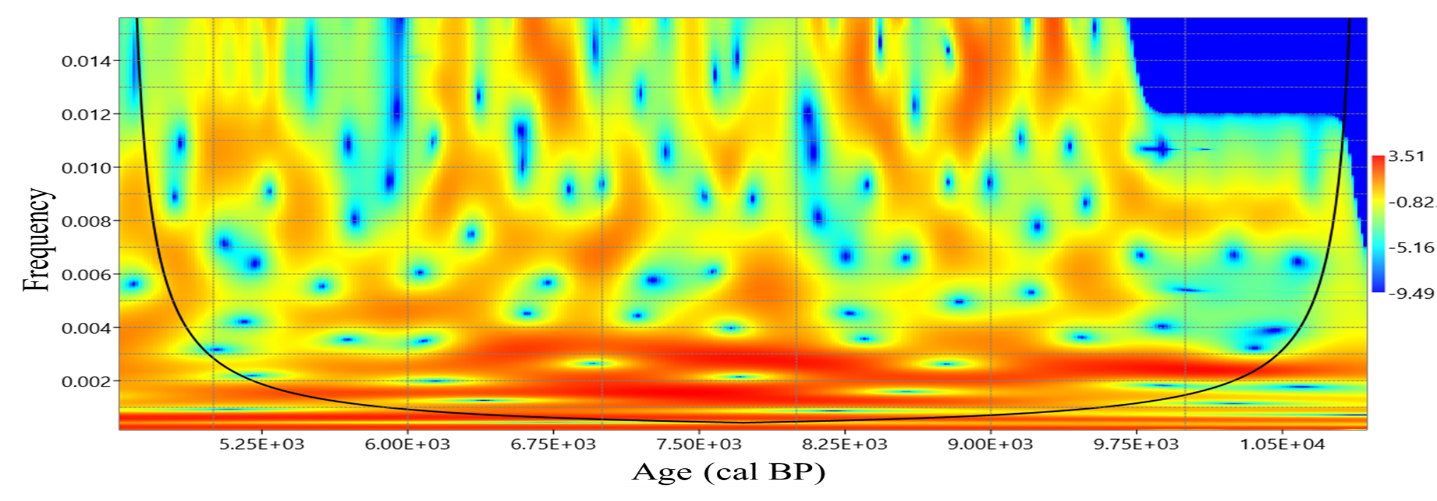


Supplementary Fig. 4. Time series of magnetic susceptibility (χlf 10^-8^ m^3^Kg^-1^) and wavelet analysis for unequally space data. The x-axis represents the time (10.9- 4.5 ka BP) while the y axis indicates frequency. The red peak indicates high magnetic susceptibility frequency. The black line indicates the cone of influence. The figure is plotted using PAST 4.0 software.

References

Hammer, Ø., Harper, D.A.T., Ryan, P.D. 2001. PAST: Paleontological statistics software package for education and data analysis. Palaeontologia Electronica 4(1): 9pp.

Stein, A.F., Draxler, R.R, Rolph, G.D., Stunder, B.J.B., Cohen, M.D., and Ngan, F., (2015). NOAA’s HYSPLIT atmospheric transport and dispersion modeling system, Bull. Amer. Meteor. Soc., **96**, 2059-2077, <http://dx.doi.org/10.1175/BAMS-D-14-00110.1>

Rolph, G., Stein, A., and Stunder, B., (2017). Real-time Environmental Applications and Display sYstem: READY. Environmental Modelling & Software, **95**, 210-228, <https://doi.org/10.1016/j.envsoft.2017.06.025>
